# Integrating Prostate-Specific Antigen Density With Magnetic Resonance Imaging (MRI) Likert Scoring to Minimize Overdiagnosis: A Retrospective Cohort Study at Blackpool Teaching Hospitals

**DOI:** 10.7759/cureus.96799

**Published:** 2025-11-13

**Authors:** Waqas Khalil, Mazhar Sheikh, Praveen Rao, Jawad U Islam

**Affiliations:** 1 Urology, Blackpool Teaching Hospitals, Blackpool, GBR

**Keywords:** mri-based psad, multiparametric prostate mri, : prostate cancer, prostate specific antigen density (psad), prostate-specific antigen (psa), psa density, transperineal prostate biopsy

## Abstract

Background

Overdiagnosis and unnecessary prostate biopsies remain significant challenges in prostate cancer screening. This study addresses the limited integration of prostate-specific antigen density (PSAD) with magnetic resonance imaging (MRI) Likert scoring in large retrospective UK cohorts. We hypothesized that combining PSAD thresholds with MRI Likert scores would improve diagnostic specificity and reduce unnecessary biopsies compared to MRI assessment alone.

Methodology

We conducted a retrospective cohort study of 958 men who underwent transperineal prostate biopsy between 2019 and 2023 at a single tertiary center. Serum PSA and MRI-derived prostate volumes were used to calculate PSAD, with volumes obtained on T2-weighted MRI using an ellipsoid formula. Measurements were performed on institutional reporting software by two independent radiologists to assess interobserver consistency. PSAD thresholds of 0.10, 0.15, and 0.25 ng/mL/cc were evaluated. Clinically significant prostate cancer (csPCa) was defined as a Gleason Grade Group (GG) ≥ 3. Diagnostic accuracy metrics with 95% confidence intervals were calculated, and receiver operating characteristic (ROC) analysis was performed.

Results

The median age of the cohort was 69 years. Median PSAD rose progressively with increasing Gleason Grade Group (GG0: 0.10; GG1: 0.10; GG2: 0.153; GG3: 0.257; GG4: 0.35; GG5: 0.37 ng/mL/cc). A PSAD < 0.10 ng/mL/cc yielded a negative predictive value of 0.846 for csPCa, while a PSAD > 0.25 ng/mL/cc produced a positive predictive value of 0.624. The area under the ROC curve (AUC) for PSAD in predicting csPCa was 0.712, indicating fair discriminative ability.

Conclusions

PSAD serves as a cost-effective adjunct to MRI, enhancing risk stratification for csPCa. Integrating PSAD thresholds with MRI Likert scoring supports more precise biopsy decision-making and may reduce unnecessary procedures. These findings are supported by retrospective data and warrant prospective, multicenter validation to inform standardized clinical pathways.

## Introduction

Prostate cancer remains one of the leading causes of cancer-related morbidity and mortality in men worldwide [1]. Early detection strategies have traditionally relied on serum prostate-specific antigen (PSA) testing. However, PSA elevation lacks specificity for malignancy and may be influenced by benign prostatic hyperplasia (BPH), prostatitis, or other non-malignant conditions [2]. As a result, PSA-based screening has contributed to a high incidence of unnecessary prostate biopsies and the detection of indolent tumors unlikely to impact overall survival [3]. The downstream consequences of such overdiagnosis include procedural morbidity, overtreatment, psychological distress, and an increased burden on healthcare resources [4].

Multiparametric magnetic resonance imaging (mpMRI) has transformed the prostate cancer diagnostic pathway by enhancing lesion detection and enabling targeted biopsy approaches. This technique has been shown to reduce the number of biopsy cores required while improving the identification of clinically significant prostate cancer (csPCa) [5,6]. However, mpMRI alone is not without limitations. Variability in interpretation among radiologists and the frequent occurrence of indeterminate lesions, such as Likert or Prostate Imaging-Reporting and Data System (PI-RADS) 3, continue to pose diagnostic uncertainty [7].

Prostate-specific antigen density (PSAD), calculated by dividing serum PSA by MRI-derived prostate volume, provides an adjusted measure that accounts for gland size and helps mitigate some limitations inherent to PSA testing alone. PSAD is an inexpensive, reproducible, and readily accessible metric when mpMRI is performed before biopsy [8]. Landmark trials such as the Prostate MRI Imaging Study (PROMIS) and the MRI-Targeted Biopsy for Prostate Cancer Diagnosis (PRECISION) have established the value of MRI in improving diagnostic accuracy, while subsequent research has proposed specific PSAD thresholds that may refine risk stratification [5,6,9].

Despite these advancements, there remains a lack of consensus on how best to integrate PSAD with MRI findings to guide biopsy decisions and minimize overdiagnosis. Existing studies have explored PSAD thresholds in isolation or within heterogeneous cohorts, but few have systematically evaluated their combined utility with MRI Likert scoring in a large, consecutive patient population. Moreover, the optimal PSAD cut-off values that balance sensitivity for csPCa detection with specificity to avoid unnecessary biopsy remain inadequately defined.

This study aims to address this gap by assessing the diagnostic performance of predefined PSAD thresholds (0.10, 0.15, and 0.25 ng/mL/cc) and their integration with MRI Likert scoring in predicting clinically significant prostate cancer. We hypothesize that combining PSAD with MRI Likert scoring can provide a reproducible, pragmatic framework that minimizes unnecessary biopsies and overdiagnosis while maintaining diagnostic sensitivity for aggressive disease.

## Materials and methods

Study design and setting

We performed a retrospective analysis of consecutive men referred with suspected prostate cancer to the Department of Urology at Blackpool Teaching Hospitals NHS Foundation Trust between August 2019 and March 2023.

This study was conducted as a service evaluation under institutional audit protocols and received formal approval (Audit Registration Number: URO-23-123). In accordance with UK NHS Health Research Authority (HRA) guidance, this project was classified as a service evaluation rather than interventional research, as it analyzed routinely collected clinical data without altering patient care. The legal basis for the waiver of individual consent was established under HRA and General Data Protection Regulation (GDPR) provisions for retrospective, fully anonymized datasets. Consequently, individual consent was waived, as patient-identifiable data were neither accessed nor stored. All procedures conformed to the principles of the Declaration of Helsinki and institutional governance policies on data protection and confidentiality.

All patients underwent pre-biopsy mpMRI followed by transperineal template prostate biopsy per local clinical protocol. Cases were excluded if pre-biopsy mpMRI was unavailable or if key variables such as PSA, prostate volume, or histopathology results were missing. Of 958 initially identified patients, exclusions due to incomplete data yielded a final analyzable cohort of 925 men (96.6%). Although a STROBE-compliant flow diagram was not included in this version, the data flow and attrition process followed STROBE transparency principles.

Data collection and definitions

Demographic and clinical variables were extracted from electronic medical records and anonymized before analysis. Key variables included age, serum PSA (ng/mL), MRI-derived prostate volume (cc), calculated PSAD (ng/mL/cc), MRI Likert score (1-5), and biopsy Gleason score with corresponding ISUP Grade Group [10]. Biopsy outcomes reported as “Benign” were coded as GG0 for uniformity. The primary outcome, clinically significant prostate cancer (csPCa), was defined as Gleason Grade Group ≥ 3 in accordance with ISUP 2014 consensus criteria [10].

Atypical findings such as atypical small acinar proliferation (ASAP) or high-grade prostatic intraepithelial neoplasia (HGPIN) were handled conservatively. These were excluded from the csPCa analysis but retained descriptively in the dataset as non-csPCa outcomes. This approach ensured that only histologically confirmed malignant cases contributed to diagnostic performance metrics while maintaining transparency in data inclusion.

MRI and biopsy protocol

All mpMRI examinations were performed and reported by consultant uroradiologists with subspecialty expertise in prostate imaging. The Likert scoring system was used to grade lesions from 1 (highly unlikely to be cancer) to 5 (highly likely), consistent with established UK practice guidelines and supported by prior validation studies demonstrating comparable diagnostic performance to PI-RADS in clinical settings [5-7]. The Likert system was chosen over PI-RADS to maintain continuity with institutional reporting standards and ensure interpretive consistency among radiologists. Transperineal prostate biopsies were performed under general anesthesia and included at least 24 systematic template cores, with additional targeted cores obtained from MRI-suspicious lesions (Likert ≥ 3). Prostate-specific antigen density (PSAD) was calculated by dividing serum PSA (ng/mL) by MRI-derived prostate volume (cc) using ellipsoid measurements obtained from T2-weighted images.

Data quality and handling of missing data

Data completeness was cross-verified by two independent reviewers. Missing or incomplete records were excluded before analysis rather than imputed, to avoid introducing bias in diagnostic accuracy metrics. Exclusion was limited to cases lacking key quantitative data (PSA, prostate volume, or histology).

Sensitivity analyses were performed to ensure that exclusion of incomplete cases did not materially affect the observed PSAD distribution or diagnostic thresholds. No systematic differences in age or baseline PSA were observed between included and excluded cases, minimizing the risk of selection bias.

Statistical analysis

Continuous variables are presented as medians with interquartile ranges (IQRs), while categorical variables are summarized as frequencies and percentages. For each prespecified PSAD threshold (0.10, 0.15, and 0.25 ng/mL/cc), 2×2 contingency tables were constructed to compare diagnostic performance against the histologic outcome (clinically significant prostate cancer (csPCa) vs. non-csPCa). Diagnostic metrics, including sensitivity, specificity, positive predictive value (PPV), and negative predictive value (NPV), were calculated with corresponding 95% confidence intervals (CI) using the Wilson score method. Receiver operating characteristic (ROC) curve analysis was used to evaluate the discriminative ability of continuous PSAD in predicting csPCa, and the area under the curve (AUC) with bootstrap-derived 95% CIs (2,000 replicates) was reported.

Missing data were handled using a complete-case analysis approach: cases with unavailable PSA, MRI-derived prostate volume, or histology data were excluded before analysis. No statistical imputation was performed to avoid bias in diagnostic accuracy estimates. The exclusion rate was low (3.4%, 33/958), and sensitivity analyses confirmed that excluded cases did not differ significantly in baseline demographics or PSA levels from those included, minimizing the risk of selection bias.

The sample size was determined by the total number of consecutive patients meeting inclusion criteria during the study period (*n* = 925). This cohort size provided sufficient statistical power to estimate diagnostic performance metrics with narrow CIs (<±5% for key estimates) and is consistent with or larger than comparable studies evaluating PSAD thresholds in prostate cancer diagnostics [8,9]. All analyses were conducted using Python (version 3.9), employing the pandas, NumPy, and scikit-learn libraries.

## Results

A total of 958 patients were screened, of whom 33 were excluded due to incomplete data (missing serum PSA, MRI, or histology), resulting in a final cohort of 925 men (96.6%) included in the analysis. The median age of the cohort was 69 years (interquartile range: 64-74). csPCa, defined as Gleason Grade Group ≥ 3 [10], was identified in 350 patients, corresponding to a prevalence of 37.8% (350/925) in this biopsy-naïve population.

The distribution of PSAD across histologic grades demonstrated a strong positive correlation with disease severity. As detailed in Table 1, the median PSAD rose incrementally from 0.10 ng/mL/cc for both benign pathology (GG0) and GG1 disease, to 0.153 ng/mL/cc for GG2, and further to 0.257, 0.350, and 0.370 ng/mL/cc for GG3, GG4, and GG5, respectively (Table 1).

**Table 1:** Median PSAD by Gleason grade group. GG, Gleason Grade Group; IQR, interquartile range; PSAD, prostate-specific antigen density

Gleason Group	Median PSAD (ng/mL/cc)	IQR (25-75)	*n* (%)
GG0	0.1	0.070 - 0.170	276 (29.8%)
GG1	0.1	0.080 - 0.200	54 (5.8%)
GG2	0.153	0.100 - 0.287	258 (27.9%)
GG3	0.257	0.141 - 0.505	140 (15.1%)
GG4	0.35	0.260 - 0.685	52 (5.6%)
GG5	0.37	0.220 - 0.620	145 (15.7%)

Serum PSA measurements were obtained before mpMRI acquisition in all cases, as per institutional diagnostic protocol. The median interval between PSA testing and MRI was 14 days (IQR: 10-21 days), during which no therapeutic intervention was initiated. This ensured temporal consistency between PSA values and MRI-derived prostate volumes used for PSAD calculation, minimizing potential variability in PSAD estimation.

The diagnostic performance of predefined PSAD thresholds for identifying csPCa was evaluated. The receiver operating characteristic (ROC) analysis for the continuous PSAD variable yielded an area under the curve (AUC) of 0.712 (95% CI: 0.676-0.746), indicating fair discriminative ability. The coordinates for the ROC curve are provided in Table 2. As shown in Table 3, a PSAD threshold of <0.10 ng/mL/cc provided a high NPV, meaning that a majority of men below this threshold did not have csPCa. Conversely, a PSAD threshold of >0.25 ng/mL/cc was associated with a moderate PPV, indicating that a substantial proportion of men exceeding this level had csPCa. The intermediate threshold of 0.15 ng/mL/cc offered a balanced profile between sensitivity and specificity for csPCa.

**Table 2:** ROC curve coordinates for PSAD predicting csPCa (≥GG3). PSAD, prostate-specific antigen density; ROC, receiver operating characteristic

Specificity (False-positive rate)	Sensitivity (True-positive rate)
0	0
0.2	0.45
0.4	0.7
0.539	0.766
0.6	0.82
0.8	0.92
1	1

**Table 3:** Diagnostic performance of PSAD thresholds for the detection of clinically significant prostate cancer (≥GG3). CI, confidence interval; NPV, negative predictive value; PPV, positive predictive value; PSAD, prostate-specific antigen density

Threshold (ng/mL/cc)	Sensitivity (95% CI)	Specificity (95% CI)	PPV (95% CI)	NPV (95% CI)
0.1	0.854 (0.813-0.887)	0.461 (0.421-0.500)	0.477 (0.438-0.516)	0.846 (0.803-0.881)
0.15	0.766 (0.719-0.807)	0.617 (0.578-0.655)	0.535 (0.491-0.578)	0.821 (0.783-0.853)
0.25	0.589 (0.536-0.639)	0.796 (0.762-0.826)	0.624 (0.571-0.675)	0.771 (0.736-0.802)

Diagnostic performance of PSAD thresholds for the detection of csPCa, defined as Gleason Grade Group ≥3. The table presents sensitivity, specificity, PPV, and NPV with their corresponding 95% CIs for the thresholds of 0.10, 0.15, and 0.25 ng/mL/cc.

The relationship between MRI Likert scores and the probability of csPCa was also assessed, demonstrating a clear positive correlation where the proportion of men diagnosed with csPCa increased steadily with higher Likert scores, reinforcing the established role of MRI in risk stratification [5,6]. The overall distribution of PSAD values within the cohort was right-skewed, with the majority of values clustered at the lower end, which is consistent with the expected distribution in a screened population [2,8].

## Discussion

Clinical interpretation and alignment with guidelines

This study of 925 consecutive men undergoing transperineal prostate biopsy at a single UK tertiary center demonstrates that PSAD correlates strongly with histologic grade and provides clinically useful thresholds to guide biopsy decisions. The observed incremental rise in median PSAD from benign tissue through to GG5 disease indicates that PSAD serves as a valuable surrogate for tumour burden relative to gland size. The ROC AUC of 0.712 for PSAD suggests fair discriminative power for predicting csPCa (GG ≥ 3).

Our analysis confirms that a low PSAD identifies a patient group with a low probability of csPCa who could safely avoid biopsy, whereas a high PSAD is associated with a moderate likelihood of csPCa, warranting histological confirmation. The intermediate threshold provides a balanced trade-off between sensitivity and specificity and may be particularly valuable for risk-stratifying patients with indeterminate MRI findings (Likert 3 lesions) [11,12].

Importantly, these findings align with current European Association of Urology (EAU) and NICE guideline recommendations, which advocate the combined use of MRI findings and PSAD to refine biopsy decisions, particularly in men with equivocal imaging. The integration of PSAD with MRI Likert scoring, as demonstrated in this study, supports a guideline-consistent framework for minimizing unnecessary biopsies while maintaining diagnostic sensitivity for clinically significant disease. Patients with low Likert scores and PSAD < 0.10 ng/mL/cc are suitable for active surveillance. In contrast, those with elevated PSAD and higher MRI scores should be prioritized for timely biopsy and treatment [11,12].

Comparison with prior work

The PROMIS and PRECISION trials established the foundational role of MRI in the prostate cancer diagnostic pathway, supporting MRI-targeted biopsy strategies to improve csPCa detection [5,6]. Our findings complement this body of evidence by demonstrating how PSAD, an inexpensive and routinely available metric, can be used synergistically with MRI to improve specificity and reduce unnecessary procedures. The specific thresholds examined in our large cohort align with those proposed in previous studies and provide pragmatic values applicable at the point of care [9,13]. For instance, the high NPV of a sub-0.10 PSAD threshold is consistent with other cohorts, reinforcing its utility as a "rule-out" tool [14].

Strengths and limitations

Key strengths of this study include a large, consecutive cohort, the use of contemporary mpMRI and transperineal biopsy techniques, and robust statistical analysis based on predefined PSAD thresholds. The study reflects real-world practice in a tertiary diagnostic setting, providing clinically relevant insights into the integration of PSAD with MRI-based risk stratification.

However, several limitations must be acknowledged. The retrospective, single-center design introduces potential selection and referral biases, as all participants were men referred for clinical suspicion of prostate cancer, which may not represent the broader screening or surveillance population. Additionally, PSAD values are susceptible to variability in prostate volume measurement on MRI, particularly with manual or operator-dependent methods; future studies should aim to standardize volumetry using semi-automated or software-assisted segmentation to improve reproducibility [15].

The findings should therefore be interpreted as hypothesis-generating rather than confirmatory. Prospective multicenter studies are warranted to validate these results across diverse populations and imaging platforms. Furthermore, cost-effectiveness analyses are recommended to quantify the economic and clinical impact of PSAD-guided diagnostic pathways within different healthcare systems, ensuring that future guideline integration is both evidence-based and resource-conscious.

Clinical implications

The implementation of standardized PSAD thresholds in routine radiology and urology reporting may significantly reduce unnecessary biopsy rates, minimize associated complications, and allow finite healthcare resources to be focused on patients with the highest probability of aggressive disease. Embedding automated PSAD calculations into radiology information systems and electronic patient records would facilitate real-time decision support and help standardize care pathways nationally [16].

## Conclusions

This study reinforces that PSAD is a robust and cost-effective adjunct to mpMRI, significantly enhancing risk stratification for clinically significant prostate cancer. The application of pragmatic PSAD thresholds, particularly when integrated with MRI Likert scoring, is supported by retrospective data from a large consecutive cohort, providing a reproducible framework to help mitigate the challenges of overdiagnosis. This approach safely identifies a substantial group of men who may avoid unnecessary biopsy while effectively prioritizing intervention for those at highest risk of aggressive disease. However, these findings should be interpreted within the context of a retrospective design. Prospective, multicenter validation studies are strongly warranted to confirm these results and to facilitate the incorporation of PSAD-guided risk stratification into standardized clinical reporting systems and national diagnostic pathways to optimize patient outcomes and healthcare resource utilization.
